# Enhancing Innovation and Creativity Amongst Trainees in Psychiatry: Linking the Clinical Practice, Academic, and Social Experiences

**DOI:** 10.1192/bjo.2022.144

**Published:** 2022-06-20

**Authors:** Lopez Okhiai, Jiann Lin Loo

**Affiliations:** Betsi Cadwaladr University Health Board, Wrexham, United Kingdom

## Abstract

**Aims:**

In the face of constant and rapid changes in the landscape of medical practices especially psychiatry, innovation and creativity are essential competencies for all trainees to remain future-proof and competent in facing the future healthcare-related challenges. Recognising this, the General Medical Council (GMC) has highlighted the need for trainees to undertake any form of quality improvement initiatives to improve patients’ care, which trainees can struggle with. This article is aimed to share the authors reflective experience on how to improve their creativity during their training in psychiatry.

**Methods:**

This is a self-study based on the authors’ personal reflections on experiences on promoting innovation and creativity in academic and non-academic work.

**Results:**

One of the beginning points of learning how to be creative is to learn from others on how to formulate a question that can be answered using research. It can be achieved by reading journals, attending conferences, and watching up-to-date webinars. By modelling others, their ideas can be translated to local practice through adaptation which essentially involves the process of innovative work. Once a person has become more adept in asking questions, deliberate observation in clinical practice helps to consolidate creativity and ideas. With an appropriate level of curiosity, everyone's experience can potentially be transformed into research questions. Effort needs be invested to review available literatures. This will help to construct a clear picture of what is available and what is the gap that has yet to be filled in, i.e., the opportunity of improvement through innovation and creativity. Working in groups allows collaborative problem-solving approaches, which is a good platform to spark new ideas. It is common to encounter obstacles and pitfalls where perseverance is crucial as a trainee can explore alternative ways of problem-solving, which again is a source of innovation.

**Conclusion:**

From the experience of the authors, a broad-based creative exploration is helpful at the initial stage and further narrowing of focus once a creative idea has taken off is important to ensure the vision of a project is achieved. Erich Fromm once said creativities requires the letting go of certainties. The core nature of psychiatry, i.e., the uncertainties is not a limitation but an opportunity to be capitalised. Rather than telling ourselves what is not possible, ask the question of “how can I do this differently”.

